# Acute *Vhl* Gene Inactivation Induces Cardiac HIF-Dependent Erythropoietin Gene Expression

**DOI:** 10.1371/journal.pone.0022589

**Published:** 2011-07-21

**Authors:** Marta Miró-Murillo, Ainara Elorza, Inés Soro-Arnáiz, Lucas Albacete-Albacete, Angel Ordoñez, Eduardo Balsa, Alicia Vara-Vega, Silvia Vázquez, Esther Fuertes, Carmen Fernández-Criado, Manuel O. Landázuri, Julián Aragonés

**Affiliations:** 1 Department of Immunology, Hospital of La Princesa, Sanitary Research Institute Princesa (IP), Autonomous University of Madrid, Madrid, Spain; 2 Animal Facility, Autonomous University of Madrid, Madrid, Spain; University of Pittsburgh, United States of America

## Abstract

Von Hippel Lindau (*Vhl*) gene inactivation results in embryonic lethality. The consequences of its inactivation in adult mice, and of the ensuing activation of the hypoxia-inducible factors (HIFs), have been explored mainly in a tissue-specific manner. This mid-gestation lethality can be also circumvented by using a floxed *Vhl* allele in combination with an ubiquous tamoxifen-inducible recombinase Cre-ER^T2^. Here, we characterize a widespread reduction in *Vhl* gene expression in Vhl^floxed^-UBC-Cre-ER^T2^ adult mice after dietary tamoxifen administration, a convenient route of administration that has yet to be fully characterized for global gene inactivation. *Vhl* gene inactivation rapidly resulted in a marked splenomegaly and skin erythema, accompanied by renal and hepatic induction of the erythropoietin (*Epo*) gene, indicative of the in vivo activation of the oxygen sensing HIF pathway. We show that acute *Vhl* gene inactivation also induced *Epo* gene expression in the heart, revealing cardiac tissue to be an extra-renal source of EPO. Indeed, primary cardiomyocytes and HL-1 cardiac cells both induce *Epo* gene expression when exposed to low O_2_ tension in a HIF-dependent manner. Thus, as well as demonstrating the potential of dietary tamoxifen administration for gene inactivation studies in UBC-Cre-ER^T2^ mouse lines, this data provides evidence of a cardiac oxygen-sensing VHL/HIF/EPO pathway in adult mice.

## Introduction

The ability of cells to respond to low O_2_ supply (hypoxia) is fundamental in numerous pathological scenarios [1]. Hypoxia-inducible transcription factors (HIF) 1α, 2α and 3α, prolyl hydroxylase domain proteins (PHDs) 1, 2 and 3 and the von Hippel-Lindau (VHL) protein are essential molecular elements in the cellular response to low O_2_ supply. In normoxia, PHDs hydroxylate prolyl residues in the HIFα subunits that are then recognized by VHL, a protein of the multiprotein E3 ubiquitin ligase complex that marks them for degradation by the proteasome [2], [3]. In conditions of hypoxia O_2_ is limited and it is insufficient to hydroxylate prolyl residues in HIFα [4], [5]. As a result, these HIFα isoforms are stabilized and form a heterodimer with the HIFβ subunit, promoting the expression of many genes involved in cellular adaptation to hypoxia [6]. This includes the expression of erythropoietin (*Epo*) in the kidney and liver in order to facilitate oxygen delivery to hypoxic tissues [7], [8], [9], [10]. Global *Vhl* gene inactivation in mice, and the ensuing HIF activation, can be used as a strategy to explore hypoxia signalling *in vivo*. However, conventional global *Vhl* gene inactivation is lethal in embryos [11], although this can be circumvented by only inducing *Vhl* gene inactivation in adult mice.

Widespread and acute gene inactivation in adult mice can be achieved through the ubiquitous expression of an inducible Cre recombinase, which can be used to eliminate the *Vhl* allelic region flanked by two loxP sites (a floxed *Vhl* allele). The nuclear activity of Cre can be induced by fusing it to a mutant form of the human estrogen receptor (ER^T2^) that does not recognize its natural ligand (17β-estradiol) at physiological concentrations but rather, it binds the synthetic estrogen receptor ligand 4-hydroxytamoxifen (4-HT) [12]. This Cre-ER^T2^ is retained in the cytoplasm and only enters the nucleus in the presence of 4-HT, where it binds to loxP sites of the corresponding floxed alleles. Like other ubiquitous promoters, widespread Cre-ER^T2^ expression can be achieved in mice using the human ubiquitin C (UBC) promoter (UBC-Cre-ER^T2^ mice) [13]. Several means of administering tamoxifen have been described in rodents, including intraperitoneal injections and gavage [13]. However, the addition of 4-HT to powdered food or drinking water is a more convenient and less stressful means of inducing Cre recombinase activity in adult mice [14], [15], [16]. While the administration of tamoxifen via drinking water is hampered by its poor solubility, its delivery in food has been successfully achieved in several mouse lines [14], [15], [16]. However, to date, the full potential of a tamoxifen diet and its efficacy in inducing global Cre-ER^T2^ activity in different organs of a Cre-ER^T2^ transgenic mouse line (e.g. UBC-Cre-ER^T2^ mice) has not been fully explored.

Here we have successfully employed diet-based tamoxifen administration, a timesaving and convenient mean of delivering tamoxifen in order to induce widespread inactivation of the *Vhl* gene in a Vhl^floxed^-UBC-Cre-ER^T2^ mouse line. After validating the efficiency of tamoxifen dietary administration, we characterized VHL/HIF oxygen-sensing dependent events that were rapidly induced by global *Vhl* inactivation *in vivo* (within just a few days) in contrast to other works that have mainly studied the *in vivo* consequences of *Vhl* gene inactivation over several weeks [17], [18]. This study validates the use of the tamoxifen diet in UBC-Cre-ER^T2^ mouse lines for global gene inactivation, and it identifies an oxygen-sensing VHL/HIF pathway controlling extra-renal *Epo* gene expression in cardiac tissue.

## Results

### Postnatal tamoxifen diet-mediated *Vhl* gene inactivation

**Figure 1:**
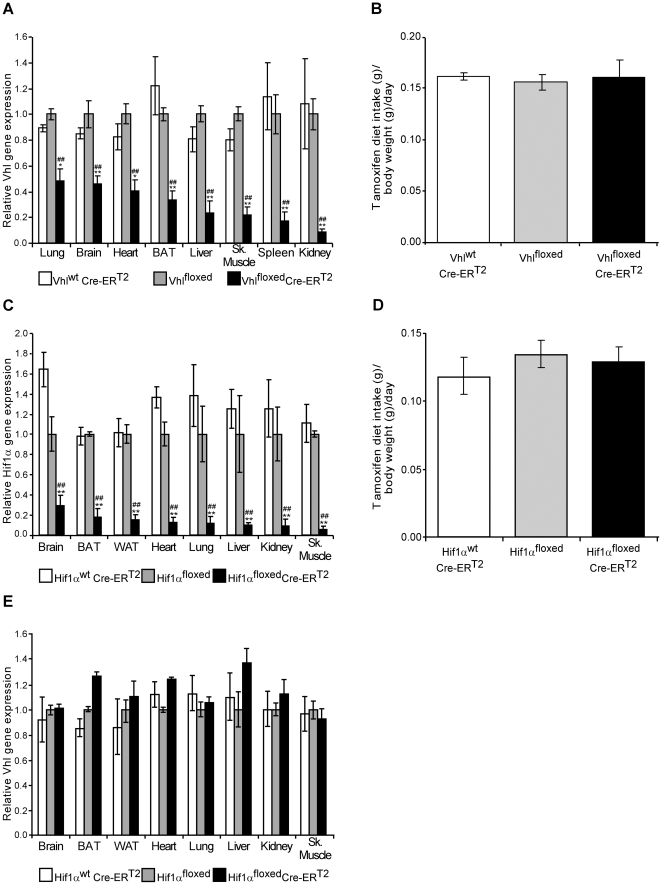
*Vhl* and *Hif1α* gene expression in tamoxifen-fed Vhl^floxed^-UBC-Cre-ER^T2^ and Hif1α^floxed^-UBC-Cre-ER^T2^ mice. (A) Vhl^wt^-UBC-Cre-ER^T2^ (n = 3), Vhl^floxed^ (n = 6) and Vhl^floxed^-UBC-Cre-ER^T2^ (n = 6) mice were placed on a tamoxifen diet for ten days followed by ten additional days on a normal diet. Gene expression was assessed by RT-PCR in the tissues indicated, the expression of the *Vhl* gene was normalized to that of *Hprt* and it was expressed as the change relative to Vhl^floxed^ mice. (B) Tamoxifen intake was measured over the 10 days of tamoxifen administration in Vhl^wt^-UBC-Cre-ER^T2^ (n = 3), Vhl^floxed^ (n = 6) and Vhl^floxed^-UBC-Cre-ER^T2^ (n = 6) mice. (C,E) Hif1α^wt^-UBC-Cre-ER^T2^ (n = 4), Hif1α^floxed^ (n = 3) and Hif1α^floxed^-UBC-Cre-ER^T2^ (n = 5) mice were administered tamoxifen as indicated above. *Hif1α* (C) or *Vhl* (E) gene expression was normalized to that of *Hprt* and expressed as the change relative to Hif1α^floxed^ mice. (D) Tamoxifen intake in Hif1α^wt^-UBC-Cre-ER^T2^ (n = 4), Hif1α^floxed^ (n = 3) and Hif1α^floxed^-UBC-Cre-ER^T2^ (n = 5) mice was measured as in B. Total intake per day was expressed relative to the body weight at the end of the tamoxifen treatment and the values represent the mean ± SEM. Statistical significance was assessed using a two-tailed Student's t-test, (*, p<0.05; **, p<0.01) when comparing Vhl^wt^-UBC-Cre-ER^T2^ or Hif1α^wt^-UBC-Cre-ER^T2^ with Vhl^floxed^-UBC-Cre-ER^T2^ or Hif1α^floxed^-UBC-Cre-ER^T2^ respectively; (^##^, p<0.01) when comparing Vhl^floxed^ or Hif1α^floxed^ with Vhl^floxed^-UBC-Cre-ER^T2^ or Hif1α^floxed^-UBC-Cre-ER^T2^ respectively.

Global *Vhl* gene inactivation results in embryonic lethality, at least in part due to placental dysfunction [11], preventing the study of the global loss of this gene in adult mice. We were interested in the short-term effects of activating the oxygen-sensing HIF pathway *in vivo*, as a result of global *Vhl* gene inactivation in adult Vhl^floxed^-UBC-Cre-ER^T2^ mice through dietary tamoxifen administration. Since the full potential of dietary tamoxifen administration for global gene inactivation has not been explored previously, we first validated the efficacy of the tamoxifen diet in reducing *Vhl* gene expression in the Vhl^floxed^-UBC-Cre-ER^T2^ mouse line. Age-matched Vhl^floxed^-UBC-Cre-ER^T2^ as well as control mice Vhl^floxed^ and Vhl^wt^-UBC-Cre-ER^T2^ were maintained for 10 days on an *ad libitum* diet of tamoxifen pellets (containing 400 mg/kg tamoxifen), before they were switched to a diet of normal chow for a further 10 days and *Vhl* gene expression was analyzed by quantitative real-time PCR in the different mouse organs. Hereinafter, the terms Vhl^floxed^, Vhl^wt^-UBC-Cre-ER^T2^ and Vhl^floxed^-UBC-Cre-ER^T2^ refer to mice that have been administered a tamoxifen diet as indicated above. The tamoxifen diet significantly reduced *Vhl* gene expression in the kidney, spleen, liver, skeletal muscle, brown adipose tissue (BAT), heart, lung and brain of Vhl^floxed^-UBC-Cre-ER^T2^ mice, reflecting widespread *Vhl* gene inactivation (Figure 1A). No differences in tamoxifen intake were observed between Vhl^floxed^-UBC-Cre-ER^T2^ and control mice (Figure 1B). Significantly, gene inactivation was not homogeneous and expression of the *Vhl* gene was more strongly downregulated in the kidney and spleen, and less so in other tissues such as the brain and lung (Figure 1A). To further validate the specificity of *Vhl* gene inactivation, we also quantified *Vhl* gene expression in another UBC-Cre-ER^T2^ system, the Hif1α^floxed^-UBC-Cre-ER^T2^ mouse line and their corresponding Hif1α^floxed^ and Hif1α^wt^-UBC-Cre-ER^T2^ control mice. While there were no significant differences in tamoxifen intake between the different lines (Figure 1D), *Hif1α* gene expression was dramatically and globally reduced, while *Vhl* gene expression was not affected in tamoxifen fed Hif1α^floxed^-UBC-Cre-ER^T2^ mice (Figure 1C, E).

**Figure 2:**
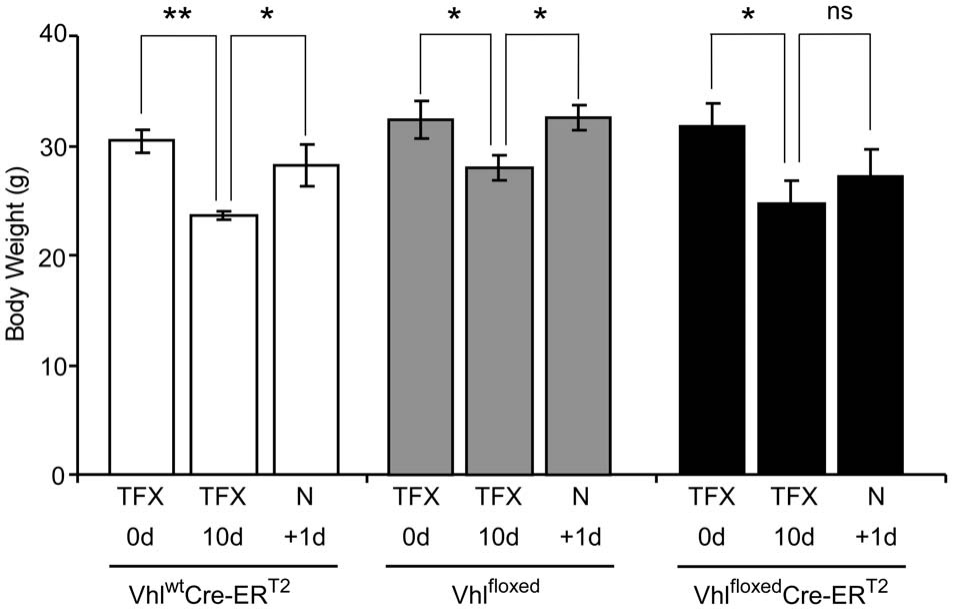
Body weight during and after tamoxifen diet administration in Vhl^floxed^-UBC-Cre-ER^T2^ and control mice. Body weight of Vhl^wt^-UBC-Cre-ER^T2^ (n = 3), Vhl^floxed^-UBC-Cre-ER^T2^ (n = 6) control Vhl^floxed^ (n = 6) mice was measured before tamoxifen treatment (TFX 0d), at the end of 10 days on a tamoxifen diet (TFX 10d) and one day after returning to a normal diet (N +1d). Statistical significance was assessed using a two-tailed Student's t-test, (*, p<0.05; **, p<0.01; ns, no significant differences).

As mice were transiently exposed to a different diet, we evaluated their body weight before and after tamoxifen treatment. Baseline body weight diminished in a similar way (∼10%) in Vhl^floxed^-UBC-Cre-ER^T2^ and control mice after 10 days on the tamoxifen diet (Figure 2). However, while the body weight of control mice returned to pre-tamoxifen levels just one day after switching back to a normal diet (Figure 2), that was not the case in Vhl^floxed^-UBC-Cre-ER^T2^ mice following tamoxifen treatment (Figure 2), suggesting that body weight was rapidly compromised by *Vhl* gene inactivation.

### Gross appearance of mice shortly after acute *Vhl* inactivation

**Figure 3:**
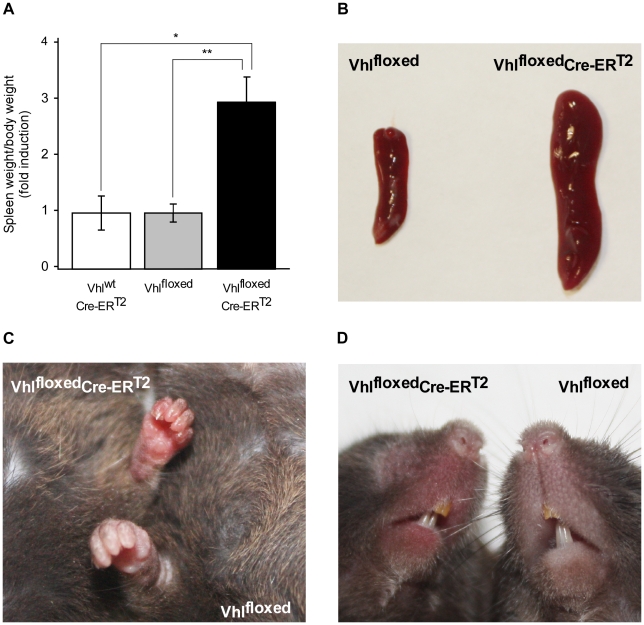
Gross appearance of tamoxifen-fed Vhl^floxed^-Cre-ERT2 mice. (A) Vhl^wt^-UBC-Cre-ER^T2^ (n = 3), Vhl^floxed^ (n = 9) and Vhl^floxed^-UBC-Cre-ER^T2^ (n = 10) mice were administered tamoxifen as indicated in Figure 1 and the spleen/body weight ratio was then determined. Statistical significance was assessed using a two-tailed Student's t-test (*, p<0.05; **, p<0.01). Representative images of spleens (B), snouts (C) and paws (D) of Vhl^floxed^-UBC-Cre-ER^T2^ and control Vhl^floxed^ mice are shown.

To further evaluate the efficacy of the tamoxifen diet on *Vhl* gene inactivation, we studied the biological consequences of acute *Vhl* inactivation soon after the mice returned to a normal diet (10 days). We evaluated spleen size and skin erythema as macroscopic indicators of activation of the HIF oxygen-sensing pathway *in vivo* [19]. All tamoxifen-treated Vhl^floxed^-UBC-Cre-ER^T2^ mice analyzed exhibited marked splenomegaly when compared with controls (Figure 3 A, B). Moreover, some mice displayed obvious reddening of their paws and snouts (Figure 3 C, D). These external signs of skin erythema appeared as early as the ninth day of tamoxifen administration (data not shown), suggesting that this phenotype represents an acute manifestation of *Vhl* gene inactivation. Overall, these data confirm that dietary administration of tamoxifen is an efficient and convenient mean to induce widespread and rapid gene inactivation of floxed alleles in UBC-Cre-ER^T2^ mice and in particular, to study the short-term biological consequences of *Vhl* inactivation.

### Acute *Vhl* inactivation induces cardiac *Epo* gene expression

**Figure 4:**
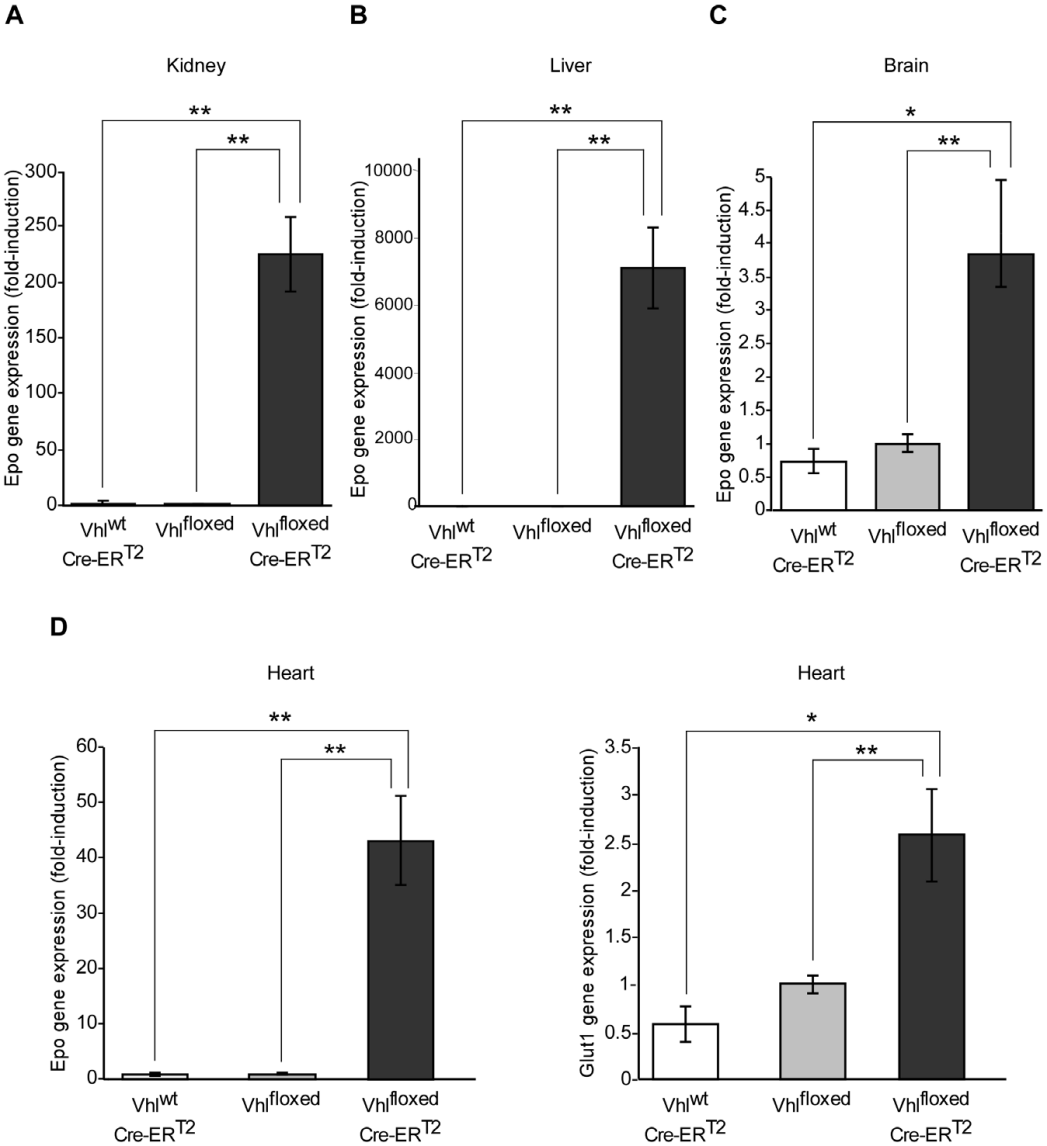
*Erythropoietin* gene expression in the kidney, liver, brain and heart of tamoxifen-fed Vhl^floxed^-UBC-Cre-ER^T2^ mice. Vhl^wt^-UBC-Cre-ER^T2^ (n = 3), Vhl^floxed^ (n = 6) and Vhl^floxed^-UBC-Cre-ER^T2^ (n = 6) mice were administered tamoxifen as indicated in Figure 1. Gene expression was assessed by RT-PCR in the kidney (A), liver (B), brain (C) and heart (D). The expression of *Epo* and *Glut1* was normalized to that of *Hprt* and expressed as the change relative to Vhl^floxed^ mice. Statistical significance was assessed using a two-tailed Student's t-test (*, p<0.05; **, p<0.01).

Splenomegaly and erythema are recognized signs of activation of the oxygen-VHL/HIF/EPO pathway, and they have been reported previously in transgenic mice overexpressing EPO [20], [21]. Given that the kidney and liver are the main sites of EPO production in adults [7], [22], [23], we investigated *Epo* gene expression in these organs in Vhl^floxed^-UBC-Cre-ER^T2^ mice shortly after *Vhl* gene inactivation. When we analyzed renal and hepatic *Epo* gene expression in tamoxifen-treated Vhl^floxed^-UBC-Cre-ER^T2^ mice, we found a strong induction of this gene in the kidney (∼200 fold, Figure 4A) and an even stronger increase in the liver when compared to control mice (Figure 4B). The marked difference between these two organs is probably due to the very low basal levels of hepatic *Epo* gene expression, which results in more marked differences when *Vhl* is inactivated. These differences cannot simply be attributed to differences in *Vhl* inactivation, as *Vhl* is inactivated to a greater extent in the kidney than in the liver (Figure 1A). Moreover, serum EPO levels were drastically elevated in tamoxifen-treated Vhl^floxed^-UBC-Cre-ER^T2^ when compared with tamoxifen-treated control mice (pg of EPO/ml: 150.5±22.6 in Vhl^floxed^ versus 49835.5±21586 in Vhl^floxed^-UBC-Cre-ER^T2^; *n* = 4, p<0.05). These mice showed a remarkable reticulocytosis. Indeed, the number of circulating reticulocytes as well as splenic reticulocytes increases in Vhl^floxed^-UBC-Cre-ER^T2^ mice (*Circulating reticulocytes x10^6^/ml* : 603.92±437 in Vhl^floxed^ versus 6391.53±1381 in Vhl^floxed^-UBC-Cre-ER^T2^; n = 3, p = 0.018) (*Splenic reticulocytes x10^6^/ml* : 32±6.08 in Vhl^floxed^ versus 269.08±4.4 in Vhl^floxed^-UBC-Cre-ER^T2^; n = 3, p = 0.01). However, a parallel hemocytometry showed that hematocrit is not significantly elevated in tamoxifen-treated Vhl^floxed^-UBC-Cre-ER^T2^ when compared with control mice (*hematocrit %*: 40.8±2.02 in Vhl^floxed^ versus 43±5.3 in Vhl^floxed^-UBC-Cre-ER^T2^; n = 5, p = NS). Furthermore, a follow up of Vhl^floxed^-UBC-Cre-ER^T2^ mice revealed that they started to show anemia after a longer time period upon *Vhl* gene inactivation (*hematocrit %*: 42.62±2.22 in Vhl^floxed^ versus 33.2±3.8 in Vhl^floxed^-UBC-Cre-ER^T2^; n = 7, p = 0.041). In addition, the proportion of Hoechst 33342^negative^ CD71^negative^ cells decrease in the spleens of Vhl^floxed^-UBC-Cre-ER^T2^ (% of total number of splenic cells: 27.10±5.3 in Vhl^floxed^ versus 3.22±1.25 in Vhl^floxed^-UBC-Cre-ER^T2^; n = 3, p<0.01). This possibly reflects a specific VHL-dependent effect on mature red blood cells survival that will be further explored in futures studies.

**Figure 5:**
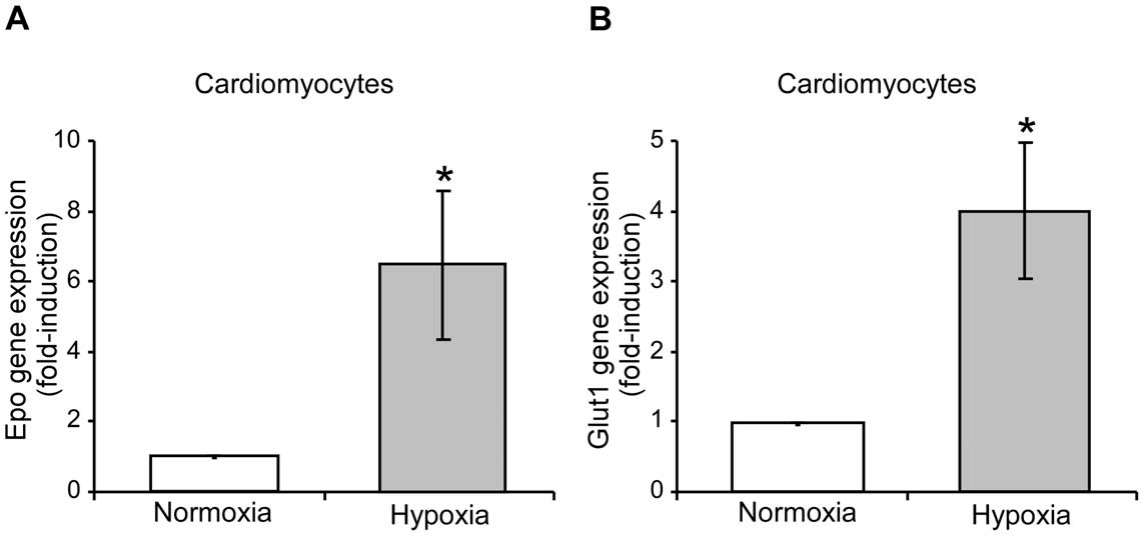
*Erythropoietin* and *glucose transporter-1* gene expression in isolated primary cardiomyocytes in response to hypoxia. Isolated rat cardiomyocyte cultures were subjected to basal normoxic conditions and/or hypoxia (1% O_2_) for 24 hours. *Epo* (A) and *Glut1* (B) expression was then analyzed by RT-PCR and normalized to that of *Hprt*. The data from four independent experiments are expressed as the change relative to the normoxic values. Statistical significance was assessed using a two-tailed paired t-test (*, p<0.05).

**Figure 6:**
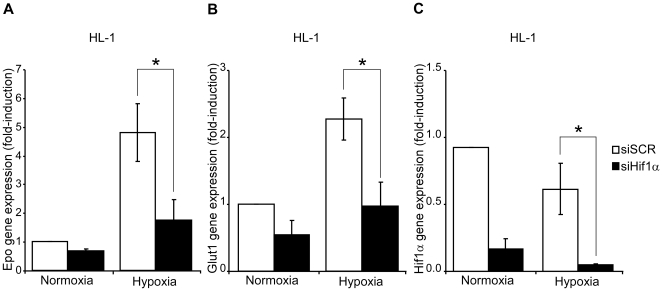
*Erythropoietin* and *glucose transporter-1* gene expression in HL-1 cardiomyocyte cell line in response to activation of the oxygen-sensing HIF pathway. (A,B,C) HL-1 cells were transfected with a siRNA for *Hif1α* (siHIF1α) or a scrambled siRNA control (siSCR) and 24 hours after transfection, the cells were exposed to normoxic or hypoxic (1% O_2_) conditions. The expression of *Epo*, *Glut1* and *Hif1α* was measured as described above and the data from three independent experiments are expressed as the change relative to the normoxic values. Statistical significance was assessed using a two-tailed Student's t-test (*, p<0.05).

In line with other studies, baseline *Epo* gene expression was particularly weak in the heart [8], although we found a remarkable elevation in cardiac *Epo* gene expression in tamoxifen fed Vhl^floxed^-UBC-Cre-ER^T2^ mice (Figure 4D). Given that cardiac *Vhl* expression is not fully ablated in tamoxifen-treated Vhl^floxed^-UBC-Cre-ER^T2^ mice (Figure 1A), we presumed that cardiac *Epo* gene expression could be potentially higher if *Vhl* deletion were more prominent. Expression of glucose transporter-1 (*Glut1)*, a HIF-dependent gene [24], was also elevated in the hearts of tamoxifen treated Vhl^floxed^-UBC-Cre-ER^T2^ mice (Figure 4D). In addition, *Epo* gene expression was markedly upregulated in the brain (Figure 4C), possibly reflecting oxygen-sensing VHL/HIF-dependent EPO production in glial cells, as described previously [25]. Induction of *Epo* gene expression was stronger in cardiac tissue than in the brain, perhaps due to the weak basal expression of the *Epo* gene in the heart. These data suggest that the oxygen-sensing VHL/HIF/EPO pathway is not restricted to classical EPO-producing tissues, and they demonstrate that the heart can express EPO upon *Vhl* inactivation. To determine whether cardiomyocytes could be contributing to this VHL-dependent response, *Epo* gene expression was analyzed in isolated primary rat cardiomyocytes exposed to low oxygen tension. While weak basal expression of the *Epo* gene was observed in normoxic cardiomyocytes, hypoxia (1% O_2_) augmented markedly its expression (Figure 5A). Likewise, *Glut1* expression was also induced, which indicates an effective induction of the HIF pathway in these experimental conditions (Figure 5B). We further evaluated the role of the HIF system in hypoxia-induced *Epo* gene expression in cardiac cells in the HL-1 cell line, a well-recognized cardiac cell model that retains a differentiated cardiac myocyte phenotype and maintains contractile activity [26]. We specifically silenced expression of *Hif1α*, the main HIF isoform expressed at RNA level in this cardiomyocyte cell line (data not shown and Figure 6C). *Glut1* expression was induced by hypoxia in control scramble-transfected HL-1 cells but its expression was markedly attenuated in HL-1 cells transfected with siHIF1α (Figure 6B). Similarly, hypoxia-induced *Epo* gene expression was reduced when *Hif1α* was silenced in these cells (Figure 6A). These data indicate that hypoxia-induced *Epo* gene expression is an autonomous VHL/HIF-dependent cardiomyocyte response that occurs shortly after activation of this oxygen-sensing pathway. This response provides a molecular and cellular explanation for the elevated levels of cardiac *Epo* gene expression in Vhl^floxed^-UBC-Cre-ER^T2^ mice.

## Discussion

The oxygen-sensing VHL/HIF dependent pathway plays a central role in cellular adaptation to oxygen fluctuations [27], [28]. This role has primarily been explored in mouse models in which HIF is chronically overactivated following tissue-specific *Vhl* inactivation [22], [25], [29]. Here, we characterize the short-term *in vivo* responses following global inactivation of *Vhl* in the Vhl^floxed^-UBC-Cre-ER^T2^ mouse line. For this purpose, we employed dietary administration of tamoxifen, a timesaving and convenient method of tamoxifen administration to induce Cre-ER^T2^ activity. The full potential for global gene inactivation was not previously explored. Indeed, dietary administration of tamoxifen has been characterized in mice with specific Cre-ER^T2^ expression in the heart, forebrain or in endothelial cells [14], [15], [16]. However, a comparative analysis of the efficiency of tamoxifen diet in different organs to determine its full potential to induce widespread gene inactivation has not been performed. Moreover, some of these studies have required several weeks on a tamoxifen diet. Here, we describe global gene inactivation in UBC-Cre-ER^T2^ mouse lines shortly (a few days) after tamoxifen administration.

In this first place, it appears that UBC-Cre-ER^T2^ is suitable to produce global gene inactivation in animals fed with a tamoxifen diet. However, tamoxifen-mediated *Vhl* gene inactivation was less pronounced in the Vhl^floxed^-UBC-Cre-ER^T2^ line than *Hif1α* gene inactivation in Hif1α^floxed^-UBC-Cre-ER^T2^ line, an effect that could not be attributed to differences in tamoxifen intake. This differential inactivation may reflect the specific efficacy of the Cre-ER^T2^ recombinase to act on the floxed region of the *Vhl* and *Hif1α* alleles. Thus, optimization of the tamoxifen diet may be necessary to achieve comparable effects in distinct UBC-Cre-ER^T2^ mouse lines. Nevertheless, the extent to which *Vhl* gene expression was reduced in these mice was sufficient to induce the activation of oxygen-sensing HIF pathways *in vivo*. Indeed, macroscopic examination of tamoxifen-treated Vhl^floxed^-UBC-Cre-ER^T2^ mice revealed marked splenomegaly, an indicator of increased activity of the oxygen-VHL/PHD/HIF sensing pathway, as seen in *Phd2* deficient and *Phd1*:*Phd3* double knock-out mice [19]. Tamoxifen-treated Vhl^floxed^-UBC-Cre-ER^T2^ mice also rapidly show signs of skin erythema (Figure 3). Indeed, reddening of the paws and snouts can be apparent as early as the ninth day of tamoxifen administration (data not shown). This could reflect an increased blood flow to the skin as a consequence of local HIF-induced nitric oxide (NO) release and subsequent local vasodilatation as has been previously shown upon chronic epidermal *Vhl* deletion [30]. Boutin et al. also show that this increased cutaneous perfusion, as a consequence of epidermal *Vhl* gene inactivation, subsequently reduces liver/skin blood flow ratio leading to elevated hepatic *Epo* gene expression [30]. However, the increased hepatic *Epo* gene expression observed in tamoxifen-treated Vhl^floxed^UBC-Cre-ER^T2^ mice is most likely a consequence of local *Vhl* gene deletion and HIF2α activation in liver.

It should be noted that other similar genetic systems have been developed to achieve inactivation of floxed alleles. Indeed, the tetracycline-dependent (Tet) system has been used for renal-specific Cre expression and subsequent inactivation of the tuberous sclerosis complex-1 (Tsc-1) when doxycycline is administered in the drinking water [31]. However, some difficulties in activating this doxycycline-dependent system in certain tissues have been reported [32], [33], [34]. By contrast, gene expression is significantly reduced in all the tissues analyzed from both Vhl^floxed^-UBC-Cre-ER^T2^ and Hif1α^floxed^-UBC-Cre-ER^T2^ mouse lines exposed to tamoxifen diet.

Tamoxifen-treated Vhl^floxed^-UBC-Cre-ER^T2^ mice have identified the heart as an additional site of EPO production upon *Vhl* inactivation. Indeed, the baseline expression of the *Epo* gene in the heart is weak but is elevated dramatically upon inactivation of *Vhl* gene expression. Experiments on isolated neonatal rat cardiomyocytes revealed that EPO upregulation is an autonomous cardiomyocyte response to hypoxia that is mediated by the oxygen-sensing VHL/HIF pathway. This response is also observed in the HL-1 cardiac cell line, an experimental model suitable to study EPO production in adult cardiac cells. HL-1 is a cardiac cell line derived from the AT-1 adult mouse atrial cardiomyocyte tumor lineage, and these cells retain a differentiated cardiac myocyte phenotype and they maintain contractile activity [26]. Moreover, erythropoietin production has been demonstrated after myocardial infarction [35], which on the basis of our data could be mediated by cardiac HIF activation.

HIF1α gene expression is higher than HIF2α in HL-1 cells, which may explain the predominant contribution of HIF1α to hypoxia-induced *Epo* gene expression in these cardiac cells. However, the relative contribution of each isoform may differ *in vivo* and indeed, immunohistological studies have identified both HIF1α and HIF2α in cardiomyocytes of mice subjected to ischemia or atmospheric hypoxia [36], [37], [38], [39], [40]. Hif1α^floxed^ mice expressing Cre driven by myosin light chain 2v (MLC2v) cardiac promoter (Hif1α^floxed^-MLC2v-Cre mice) markedly reduced HIF1α mRNA and protein expression in the heart, providing genetic evidence of *Hif1α* gene expression in cardiomyocytes [36]. Several studies have demonstrated a critical role for HIF1α in multiple cardiac oxygen-sensing pathways *in vivo* [29], [36], [41]. Thus, HIF1α could potentially drive cardiac *Epo* gene expression upon *Vhl* gene inactivation. However, HIF2α is the main contributor to HIF-induced *Epo* gene expression upon *Vhl* gene inactivation in the kidney, liver and glial cells [22], [25], [42], [43]. Further studies will therefore be required to assess the relative contribution of these isoforms *in vivo*, and especially that of HIF2α to VHL/HIF-dependent cardiac EPO expression. It should be also noted that HIF1α and HIF2α are also found in cardiac stromal cells. Indeed, cardiac endothelial cells abundantly express both HIF isoforms when oxygen supply to myocardium becomes limited, as do cells in the vessel wall that are presumably smooth muscle cells, [36], [37], [38], [39], [40]. Therefore, cardiac *Epo* gene expression upon *Vhl* gene inactivation involves HIF activation in cardiomyocytes, although we cannot rule out the involvement of HIF activation in other cardiac cell types.

Elevation of cardiac *Epo* gene expression is very remarkable, although it occurs to a lesser extent than in the liver and kidney. Therefore, it is conceivable that cardiac EPO production serves a local autocrine or paracrine function when oxygen supply to cardiac tissue becomes limited. Indeed, several studies have shown that EPO protects cardiac tissue during ischemia and the ischemia-reperfusion insult, particularly by overactivating the serine threonine kinase AKT, as well as through other pathways involving sonic hedgehog [44], [45], [46]. Indeed, the myocardium of patients undergoing bypass is protected when pyruvate, a previously recognized suppressor of PHD activity, is used [47], which correlates with a remarkable upregulation of *Epo* gene expression [48]. However, the effect of pyruvate on *Epo* gene expression was not directly assessed in cardiac cells, nor was the direct contribution of HIF activity, as we have studied in this work. Furthermore, cardiac tolerance to ischemic damage induced by ischemic preconditioning in the heart involves HIF1α mediated upregulation of key cardioprotective molecules, such as ecto-5′-nucleotidase CD73 that generates adenosine, and the A2B adenosine receptor (A2BAR) [49]. Therefore, the cardiac oxygen-sensing VHL/HIF/EPO pathway may represent an endogenous cardioprotective response that works in tandem with other pathways (e.g. adenosine) to locally induce cardiomyocyte tolerance against ischemia or ischemia-reperfusion damage.

## Materials and Methods

### Ethics Statement

All the experimental procedures were approved by the Research Ethics Committee at the UAM (Autonomous University of Madrid) and they were carried out under the supervision of the Head of Animal Welfare and Health at the UAM in accordance with Spanish and European guidelines (B.O.E, 18 March 1988, and 86/609/EEC European Council Directives).

### Cell culture and hypoxic conditions

The murine HL-1 cardiac cell line was cultured in Claycomb medium [26] containing 10% heat-inactivated Fetal Bovine Serum (FBS: Cambrex) and supplemented with 0.1 mM norepinephrine (Sigma) and 2 mM GLUTAMAX-I (Invitrogen). Cells were plated on gelatin (Difco) and fibronectin (Sigma) precoated surfaces, and cultured at 37°C for 16 hours. Neonatal rat cardiomyocytes were isolated from the hearts of 1 day-old Wistar rats using the Neomyt isolation system (Cellutron Life Technologies). To remove contaminating cardiac fibroblasts, dissociated cells were pre-plated for 1 hour on uncoated culture plates. The resulting suspension of cardiomyocytes was plated (2–3 million cells/60 mm plate) and cultured for 24 hours in medium supplemented with 10% FBS and 10 mM 5-bromo-2′-deoxyuridine (BrdU; Sigma, B5002), and then for an additional 24 hours in serum-free conditions. The cells were subjected to hypoxia in DMEM + 10% FBS. All media were supplemented with 100 U/ml penicillin, 100 µg/ml streptomycin and 1% HEPES buffer. Normoxic cells (21% O_2_) were maintained at 37°C in an incubator with 5% CO_2_. To induce hypoxia, cell culture dishes were placed into an Invivo_2_ 400 humidified hypoxia workstation (Ruskinn Technologies, Bridgend, UK) with 1% O_2_.

### Mice

C;129S-*Vhlh^tm1Jae^*/J mice (Jackson Laboratories, stock no. 4081) were used to generate the Vhl^floxed^-UBC-Cre-ER^T2^ mice. These mice harbor two loxP sites flanking the promoter and exon 1 of the murine *Vhl* locus [50]. C;129S-*Vhlh^tm1Jae^*/J mice were crossed with B6.Cg-Tg(UBC-Cre/ER^T2^)1Ejb/J mice (Jackson Laboratories, stock no. 008085) which ubiquitously express a tamoxifen-inducible Cre recombinase (Cre-ER^T2^), [13]. Vhl^floxed^-UBC-Cre-ER^T2^ mice were generated through the appropriate crosses, along with the corresponding controls, Vhl^wt^-UBC-Cre-ER^T2^ and Vhl^floxed^. Hif1α^floxed^-UBC-Cre-ER^T2^ mice were generated from B6.129-Hif1a^tm3Rsjo^/J mice (Jackson Laboratories, stock no. 007561), which harbor two loxP sites flanking exon 2 of the murine *Hif1α* locus [51]. These mice were crossed with Tg(UBC-Cre/ER^T2^)1Ejb/J mice as described above to generate Hif1α^floxed^-UBC-Cre-ER^T2^ mice and their corresponding controls, Hif1α^wt^-UBC-Cre-ER^T2^ and Hif1α^floxed^ mice.

The mice were bred and housed in a specific pathogen free (SPF) animal area of the animal facility at the Autonomous University of Madrid (UAM). For gene inactivation, Vhl^wt^-UBC-Cre-ER^T2^, Hif1α^wt^-UBC-Cre-ER^T2^ and the corresponding control males (10–5 weeks old) were fed *ad libitum* for ten days with Teckland CRD TAM^400^/CreER tamoxifen pellets (Harlan Teklad), which contain 400 mg tamoxifen citrate/kg. Subsequently, they were returned to a diet of standard mouse chow (Safe®, Augy, France) for an additional 10 days.

### Reticulocyte counts and hematocrit measurement

The number of circulating or splenic reticulocytes was determined by counting total blood or splenic cells respectively followed by a flow cytometry analysis to determine the proportion of reticulocytes identified as CD71 positive cells (using the anti-CD71-PE, Beckton-Dickinson) and low intracellular nucleic acid content (using the DNA dye Hoechst 33342) [52]. Similarly, splenic mature erythrocytes were identified as CD71 negative and Hoechst 33342 negative cells [52]. Hematocrit measurements were performed using a hemocytometer (apparatus SYSMEX KX-21N).

### Quantitative real-time PCR analysis and primers

Mice were anaesthetized by intraperitoneal administration of ketamine (Ketolar® 50 mg/ml) and xylazine (Rompun® 20 mg/ml), and the tissues of interest were then removed and snap-frozen in liquid nitrogen. Subsequently, the tissue was homogenized in Trizol (Invitrogen) with two freeze/thaw cycles and total RNA was isolated using the RNeasy RNA extraction kit (Qiagen). cDNA was prepared by reverse transcription of RNA (1 µg) using Improm-II reverse transcriptase (Promega), and polymerase chain reaction (PCR) amplification was performed using a Power SYBR Green PCR Master Mix kit (Applied Biosystems). The following primer sets were used: mouse VHL (forward, 5′-TCAGCCCTACCCGATCTTACC-3′; reverse, 5′-ATCCCTGAAGAGCC AAAGATGA-3′); mouse HIF-1α (forward, 5′-CACCGATTCGCCATGGA-3′; reverse, 5′-TCGACGTTCAGAACTCATCTTTTT-3′); rat HIF-1α (forward, 5′-GTCCTGTGGTGACTTGTCCTT-3′; reverse, 5′-TGGACTC TGATCATCTGACCAAA-3′); mouse erythropoietin (EPO) (forward, 5′-TCATCTGCGACAGTCGAGTTCT-3′; reverse, 5′-TTTTCACTCAGTCTGGGACCTTCT-3′); rat erythropoietin (EPO) (forward, 5′-CAAGGAGGCAGAAAATGTCACA-3′; reverse, 5′-TTTCCAAGCGTAG AAGTTGACTTTG-3′); mouse glucose transporter 1 (GLUT1) (forward, 5′-CGCAACGAGGAGAACC-3′; reverse, 5′-GCCGTG TTGACGATACC-3′); hypoxanthine-guanine phosphoribosyltransferase (HPRT) (forward, 5′-GTTAAGCAGTACAGCCCCAAA-3′; reverse, 5′-AGGGCATATCCAACAAC AAACTT-3′). The data were analyzed using StepOne Software version 2.0 (Applied Biosystems).

### siRNA transfection

HL-1 cells were transfected with a siRNA targeting mouse HIF-1α (50 nM, sc-44225: Santa Cruz) or a scrambled control siRNA (sc-37007), using Lipofectamine 2000 (Invitrogen) according to the manufacturer's instructions. 24 hours after transfection the cells were exposed to normoxic or hypoxic conditions for an additional 24 hours.

### Statistical analysis

The data are presented as the mean ± SEM. Statistical significance was assessed using a two-tailed Student's t-test in all figures, except in Figures 6A and B in which a two-tailed paired t-test was used.

## References

[1] Giaccia A, Siim BG, Johnson RS (2003). HIF-1 as a target for drug development.. Nat Rev Drug Discov.

[2] Ivan M, Kondo K, Yang H, Kim W, Valiando J (2001). HIFalpha targeted for VHL-mediated destruction by proline hydroxylation: implications for O2 sensing.. Science.

[3] Jaakkola P, Mole DR, Tian YM, Wilson MI, Gielbert J (2001). Targeting of HIF-alpha to the von Hippel-Lindau ubiquitylation complex by O2-regulated prolyl hydroxylation.. Science.

[4] Bruick RK, McKnight SL (2001). A conserved family of prolyl-4-hydroxylases that modify HIF.. Science.

[5] Epstein AC, Gleadle JM, McNeill LA, Hewitson KS, O'Rourke J (2001). C. elegans EGL-9 and mammalian homologs define a family of dioxygenases that regulate HIF by prolyl hydroxylation.. Cell.

[6] Semenza GL (2007). Hypoxia-inducible factor 1 (HIF-1) pathway.. Sci STKE.

[7] Eckardt KU, Kurtz A (2005). Regulation of erythropoietin production.. Eur J Clin Invest.

[8] Fandrey J, Bunn HF (1993). In vivo and in vitro regulation of erythropoietin mRNA: measurement by competitive polymerase chain reaction.. Blood.

[9] Goldberg MA, Imagawa S, Strair RK, Bunn HF (1991). Regulation of the erythropoietin gene in Hep 3B cells.. Semin Hematol.

[10] Imagawa S, Goldberg MA, Bunn HF (1989). Regulation of the erythropoietin gene.. Adv Exp Med Biol.

[11] Gnarra JR, Ward JM, Porter FD, Wagner JR, Devor DE (1997). Defective placental vasculogenesis causes embryonic lethality in VHL-deficient mice.. Proc Natl Acad Sci U S A.

[12] Feil R, Wagner J, Metzger D, Chambon P (1997). Regulation of Cre recombinase activity by mutated estrogen receptor ligand-binding domains.. Biochem Biophys Res Commun.

[13] Ruzankina Y, Pinzon-Guzman C, Asare A, Ong T, Pontano L (2007). Deletion of the developmentally essential gene ATR in adult mice leads to age-related phenotypes and stem cell loss.. Cell Stem Cell.

[14] Casanova E, Fehsenfeld S, Lemberger T, Shimshek DR, Sprengel R (2002). ER-based double iCre fusion protein allows partial recombination in forebrain.. Genesis.

[15] Forde A, Constien R, Grone HJ, Hammerling G, Arnold B (2002). Temporal Cre-mediated recombination exclusively in endothelial cells using Tie2 regulatory elements.. Genesis.

[16] Kiermayer C, Conrad M, Schneider M, Schmidt J, Brielmeier M (2007). Optimization of spatiotemporal gene inactivation in mouse heart by oral application of tamoxifen citrate.. Genesis.

[17] Ma W, Tessarollo L, Hong SB, Baba M, Southon E (2003). Hepatic vascular tumors, angiectasis in multiple organs, and impaired spermatogenesis in mice with conditional inactivation of the VHL gene.. Cancer Res.

[18] Young AP, Schlisio S, Minamishima YA, Zhang Q, Li L (2008). VHL loss actuates a HIF-independent senescence programme mediated by Rb and p400.. Nat Cell Biol.

[19] Takeda K, Aguila HL, Parikh NS, Li X, Lamothe K (2008). Regulation of adult erythropoiesis by prolyl hydroxylase domain proteins.. Blood.

[20] Heinicke K, Baum O, Ogunshola OO, Vogel J, Stallmach T (2006). Excessive erythrocytosis in adult mice overexpressing erythropoietin leads to hepatic, renal, neuronal, and muscular degeneration.. Am J Physiol Regul Integr Comp Physiol.

[21] Vogel J, Kiessling I, Heinicke K, Stallmach T, Ossent P (2003). Transgenic mice overexpressing erythropoietin adapt to excessive erythrocytosis by regulating blood viscosity.. Blood.

[22] Rankin EB, Biju MP, Liu Q, Unger TL, Rha J (2007). Hypoxia-inducible factor-2 (HIF-2) regulates hepatic erythropoietin in vivo.. J Clin Invest.

[23] Zanjani ED, Ascensao JL, McGlave PB, Banisadre M, Ash RC (1981). Studies on the liver to kidney switch of erythropoietin production.. J Clin Invest.

[24] Chen C, Pore N, Behrooz A, Ismail-Beigi F, Maity A (2001). Regulation of glut1 mRNA by hypoxia-inducible factor-1. Interaction between H-ras and hypoxia.. J Biol Chem.

[25] Weidemann A, Kerdiles YM, Knaup KX, Rafie CA, Boutin AT (2009). The glial cell response is an essential component of hypoxia-induced erythropoiesis in mice.. J Clin Invest.

[26] White SM, Constantin PE, Claycomb WC (2004). Cardiac physiology at the cellular level: use of cultured HL-1 cardiomyocytes for studies of cardiac muscle cell structure and function.. Am J Physiol Heart Circ Physiol.

[27] Mole DR, Ratcliffe PJ (2008). Cellular oxygen sensing in health and disease.. Pediatr Nephrol.

[28] Semenza GL (2002). Involvement of hypoxia-inducible factor 1 in human cancer.. Intern Med.

[29] Lei L, Mason S, Liu D, Huang Y, Marks C (2008). Hypoxia-inducible factor-dependent degeneration, failure, and malignant transformation of the heart in the absence of the von Hippel-Lindau protein.. Mol Cell Biol.

[30] Boutin AT, Weidemann A, Fu Z, Mesropian L, Gradin K (2008). Epidermal sensing of oxygen is essential for systemic hypoxic response.. Cell.

[31] Traykova-Brauch M, Schonig K, Greiner O, Miloud T, Jauch A (2008). An efficient and versatile system for acute and chronic modulation of renal tubular function in transgenic mice.. Nat Med.

[32] Hochedlinger K, Yamada Y, Beard C, Jaenisch R (2005). Ectopic expression of Oct-4 blocks progenitor-cell differentiation and causes dysplasia in epithelial tissues.. Cell.

[33] Hsiao EC, Nguyen TD, Ng JK, Scott MJ, Chang WC (2011). Constitutive Gs activation using a single-construct tetracycline-inducible expression system in embryonic stem cells and mice.. Stem Cell Res Ther.

[34] Katsantoni EZ, Anghelescu NE, Rottier R, Moerland M, Antoniou M (2007). Ubiquitous expression of the rtTA2S-M2 inducible system in transgenic mice driven by the human hnRNPA2B1/CBX3 CpG island.. BMC Dev Biol.

[35] Mengozzi M, Latini R, Salio M, Sfacteria A, Piedimonte G (2006). Increased erythropoietin production after myocardial infarction in mice.. Heart.

[36] Huang Y, Hickey RP, Yeh JL, Liu D, Dadak A (2004). Cardiac myocyte-specific HIF-1alpha deletion alters vascularization, energy availability, calcium flux, and contractility in the normoxic heart.. Faseb J.

[37] Jurgensen JS, Rosenberger C, Wiesener MS, Warnecke C, Horstrup JH (2004). Persistent induction of HIF-1alpha and -2alpha in cardiomyocytes and stromal cells of ischemic myocardium.. Faseb J.

[38] Kim CH, Cho YS, Chun YS, Park JW, Kim MS (2002). Early expression of myocardial HIF-1alpha in response to mechanical stresses: regulation by stretch-activated channels and the phosphatidylinositol 3-kinase signaling pathway.. Circ Res.

[39] Stroka DM, Burkhardt T, Desbaillets I, Wenger RH, Neil DA (2001). HIF-1 is expressed in normoxic tissue and displays an organ-specific regulation under systemic hypoxia.. Faseb J.

[40] Wiesener MS, Jurgensen JS, Rosenberger C, Scholze CK, Horstrup JH (2003). Widespread hypoxia-inducible expression of HIF-2alpha in distinct cell populations of different organs.. Faseb J.

[41] Cai Z, Zhong H, Bosch-Marce M, Fox-Talbot K, Wang L (2008). Complete loss of ischaemic preconditioning-induced cardioprotection in mice with partial deficiency of HIF-1 alpha.. Cardiovasc Res.

[42] Kapitsinou PP, Liu Q, Unger TL, Rha J, Davidoff O (2010). Hepatic HIF-2 regulates erythropoietic responses to hypoxia in renal anemia.. Blood.

[43] Warnecke C, Zaborowska Z, Kurreck J, Erdmann VA, Frei U (2004). Differentiating the functional role of hypoxia-inducible factor (HIF)-1alpha and HIF-2alpha (EPAS-1) by the use of RNA interference: erythropoietin is a HIF-2alpha target gene in Hep3B and Kelly cells.. Faseb J.

[44] Camici GG, Stallmach T, Hermann M, Hassink R, Doevendans P (2007). Constitutively overexpressed erythropoietin reduces infarct size in a mouse model of permanent coronary artery ligation.. Methods Enzymol.

[45] Burger D, Xenocostas A, Feng QP (2009). Molecular basis of cardioprotection by erythropoietin.. Curr Mol Pharmacol.

[46] Ueda K, Takano H, Niitsuma Y, Hasegawa H, Uchiyama R (2010). Sonic hedgehog is a critical mediator of erythropoietin-induced cardiac protection in mice.. J Clin Invest.

[47] Lu H, Dalgard CL, Mohyeldin A, McFate T, Tait AS (2005). Reversible inactivation of HIF-1 prolyl hydroxylases allows cell metabolism to control basal HIF-1.. J Biol Chem.

[48] Ryou MG, Flaherty DC, Hoxha B, Sun J, Gurji H (2009). Pyruvate-fortified cardioplegia evokes myocardial erythropoietin signaling in swine undergoing cardiopulmonary bypass.. Am J Physiol Heart Circ Physiol.

[49] Eckle T, Kohler D, Lehmann R, El Kasmi K, Eltzschig HK (2008). Hypoxia-inducible factor-1 is central to cardioprotection: a new paradigm for ischemic preconditioning.. Circulation.

[50] Haase VH, Glickman JN, Socolovsky M, Jaenisch R (2001). Vascular tumors in livers with targeted inactivation of the von Hippel-Lindau tumor suppressor.. Proc Natl Acad Sci U S A.

[51] Ryan HE, Lo J, Johnson RS (1998). HIF-1 alpha is required for solid tumor formation and embryonic vascularization.. Embo J.

[52] Chen SY, Wang Y, Telen MJ, Chi JT (2008). The genomic analysis of erythrocyte microRNA expression in sickle cell diseases.. PLoS One.

